# Dietary vitamin A intakes of chinese children with adequate liver stores as assessed by the retinol isotope dilution technique

**DOI:** 10.1186/s12887-022-03660-0

**Published:** 2022-10-17

**Authors:** Jing Zeng, Yanming Li, Yan Ren, Weiwei Gu, Zhaolin Li, Mei Yang, Bing Xiang

**Affiliations:** 1 School of Public Health, Wuhan University of Science and Technology, 430065 Wuhan, Hubei China; 2 Shiyan Centre for Disease Control and Prevention, 442000 Shiyan, Hubei China; 3 Research Center for Health Promotion in Women, Youth and Children, Wuhan University of Science and Technology, 430065 Wuhan, Hubei China

**Keywords:** Vitamin A, Deuterated-retinol-dilution, Dietary requirement, Children, Adequate intake

## Abstract

**Background:**

We attempted to estimate dietary vitamin A requirements based on dietary vitamin A intake in well-nourished Chinese children with adequate liver vitamin A reserves.

**Methods:**

A cross-sectional study was conducted in a kindergarten and an elementary school in Shiyan city, Hubei province of China from December 2009 to July 2010. After screening, 60 children (22 aged 4 ~ 6 y and 38 aged 7 ~ 9 y) were randomly subjected to a 3-d or 18-d deuterated-retinol-dilution (DRD) procedure to evaluate the vitamin A reserves in the body and liver. Dietary intakes of vitamin A were estimated from two (one in winter and one in summer) consecutive 3-day weighed food records and dietary recalls.

**Results:**

The dietary vitamin A intakes were significantly correlated with vitamin A stores in the body and liver, but not with the serum level of retinol. The dietary vitamin A intakes were 476.9 ± 196.7 µg retinol equivalent (RE) (or 377.7 ± 166.2 µg retinol activity equivalent (RAE)) / day for 4 ~ 6 y children and 529.1 ± 87.2 µg RE/d (or 464.0 ± 81.1 µg RAE/d) for 7 ~ 9 y children with adequate liver vitamin A reserves. The estimated liver stores of vitamin A derived from both time points (3-d and 18-d) were similar.

**Conclusion:**

Adequate dietary vitamin A intakes among the well-nourished Chinese children were estimated to be 477 µg RE/d (95%CI 385 ~ 570) or 378 µg RAE/d (95%CI 304 ~ 441) for 4 ~ 6 y children and 529 µg RE/d (95%CI 500 ~ 560) or 464 µg RAE/d (95%CI 437 ~ 491) for 7 ~ 9 y children. Although it needs to be verified in a larger population of different regions in China, our results provide important data to establish the dietary requirement of vitamin A specifically for Chinese children.

**Supplementary Information:**

The online version contains supplementary material available at 10.1186/s12887-022-03660-0.

## Background

Vitamin A is essential for normal vision, growth, cellular differentiation and proliferation, reproduction, and immune responses. Children are particularly vulnerable to vitamin A deficiency (VAD). According to the World Health Organization (WHO) estimates, 190 million (33.3%) preschool-age children are vitamin A deficient, with the highest prevalence in developing countries in Africa and Southeast Asia [1]. It has been reported that the prevalence of VAD (serum retinol < 0.7µmol/L) was 8.18%, and marginal VAD (serum retinol 0.7 ~ 1.05 µmol/L) was 21.8% among Chinese children aged 6 to 13 years [2]. So, VAD is still a public health problem among Chinese children.

Unfortunately, since there is no metabolic study for Chinese children and adolescents, the appropriate amount of vitamin A intake specifically recommended for Chinese children has not been established. The current Chinese Recommended Nutrient Intakes (RNIs) of vitamin A for children (RNIs: 360 µg RAE/d for the children aged 4–6 y, and 500 µg RAE/d for the children aged 7–11 y) [3] were established by referring to the data of Chinese adults (RNIs: 700 µg RAE/d for the women, and 800 µg RAE/d for the men.) [3], which was estimated based on the Olson Eq. [4] and Wang’s study on the evaluation of vitamin A requirement by isotope dilution technique and vitamin A intervention experiment in Chinese adults [5]. But the current recommendation may or may not be appropriate for Chinese children for the different diet between adults and children.

Adequate intake (AI) is a recommended average daily nutrient intake level, based on experimentally derived intake levels or approximations of observed mean nutrient intake by a group (or groups) of apparently healthy people that are assumed to be adequate [6, 7]. Most of the body’s vitamin A reserve remains in the liver, thus liver concentration of vitamin A is a gold standard of vitamin A status [8, 9]. The concentration of vitamin A in liver ≥ 0.07µmol/g, which could meet all physiological needs and maintain a reserve for 3 ~ 4 months when intakes are low or the needs increase, was used as a cut-off criterion for vitamin A adequacy [4]. Direct measurement of hepatic vitamin A is not feasible under normal circumstances, and the retinol isotope dilution (RID) technique provides a quantitative estimate of total-body vitamin A and liver vitamin A stores without directly measuring liver samples [10–15]. Several studies have used RID technique successfully to evaluate vitamin A status for the elderly [16, 17], adults [18–20], and children [21–27], but few studies were carried out on dietary requirement for children. Conventional DRD procedure needs 11 ~ 26 days of equilibration between serum deuterated vitamin A and the body’s vitamin A pool after dosing of deuterated vitamin A [10–15]. A shorter term (such as 3 days) DRD provides the possibility to predict total body stores of vitamin A instead of conventional DRD and is more practical in field settings, but this procedure needs to be further verified [28–30].

Therefore, the first objective of the study was to estimate the amount of daily vitamin A intake required to maintain an adequate liver reserve for Chinese children. Meanwhile, the research can also assess whether 3-d DRD technique could be used to estimate the stores of vitamin A in the present study.

## Materials and methods

### Subjects

The field study was carried out in a kindergarten and an elementary school in Shiyan city, Hubei province of China from December 2009 to July 2010. The kindergarten and elementary school were selected because they represented communities with middle socioeconomic status and had complete health records for each child. Initially, 101 kindergarten children (4–6 y) and 102 elementary school children (7–9 y) were subjected to a questionnaire survey on personal information, medical history and dietary habits including dietary supplements. Then, the anthropometric measurements and clinical examinations were performed by trained pediatrician from the People’s Hospital of Shiyan. Fecal samples were collected for fat absorption and parasites infection test. Blood samples (3 ~ 5 ml) were collected for measurements of blood cell count, hemoglobin, hematocrit, albumin, C-reactive protein (CRP), and concentration of serum retinol.

Children with the following conditions were excluded: ongoing acute or chronic disease, having taken nutritional supplements within 3 months, positive results in the fat absorption test or parasite test, increased level of CRP (> 8 mg/L), or lower serum level of retinol (< 1.05 µmol/L). Among the subjects, 6 subjects (3.0%) had lower serum level of retinol (< 0.7µmol/L), which indicate vitamin A deficiency; and 27 subjects (13.6%) had serum retinol levels (0.7µmol/L ~ 1.05µmol/L) that marginally adequate. After the screening, 123 children were selected and completed the study, but 60 subjects with higher serum level of retinol were randomly selected for the DRD test. There was no statistically significant difference in the age, gender, health status, and serum retinol concentration of the two groups of children, an additional file showed this in more detail [see Additional file 1].The data presented in this paper only comes from these 60 subjects, the flow diagram of subject recruitment is shown in an additional file [see Additional file 2].

### Processing of blood samples

Venous blood was drawn from the forearm of each child by venipuncture. Each blood sample was collected in 2 tubes with 3 ~ 5ml in each tube. One tube was sent to the People’s Hospital of Shiyan immediatetly to determine blood cell number and levels of hemoglobin, hematocrit, albumin, and CRP. Blood cell count, levels of hemoglobin and hematocrit, were assayed by Abbott CD-3200 automatic biochemical analyzer (Abbott Laboratories, USA). Serum levels of albumin were determined by using a commercial albumin assay kit (Zhejiang Kuake Biotechnology LTD, China). The other tube of blood sample was set at room temperature (25℃) for 0.5 h., centrifuged at 1000 rpm for 15 min, then divided into aliquots (0.5 ~ 1ml) and transferred into cryovials that were immediately stored at -20℃. At the end of field work, the samples were transported under dry ice to Tongji Medical School and stored at -80℃ until used to determine the level of serum retinol and carotenoids. Serum retinol and carotenoids were extracted with chloroform : methanol (2 : 1, vol : vol) and hexane, then analyzed by gradient reversed-phase HPLC at 340 and 450 nm with a C30 column [31].

### Dietary survey

Dietary surveys were carried out twice, one in winter (December 2009) and another in summer (July 2010), to reflect the effect of season change. There was statistically significant differences between the two seasons, an additional file showed this in more detail [see Additional file 3]. For the kindergarten children, the dietary surveys include two parts: the food intake in the school and the food intake at home. The elementary school was a boarding school, so the children in the elementary school ate only in the school from Monday morning to Friday afternoon. The amount and components of diets (including snacks) for all children in the kindergarten or elementary school were estimated by two consecutive 3-day weighed food records, a total of 6 days. Specifically, all raw materials and ingredients of every course were weighed and recorded before and after they were cooked in order to calculate the coefficients/ratios of the raw materials to cooked foods in each course dish. The amount of each course consumed by each child per meal and the leftovers were weighed, recorded, and then translated into the amount of raw materials which taken by each child by using the coefficients described above. Investigators can participate in the survey only after receiving strict and professional training and passing the examination, unified calibration scales were used for food weighing, so as to minimize the errors caused by investigators and instruments.

The food intakes of kindergarten children at home for the 6-day records approach were estimated by dietary recall. Specifically, the amounts and kinds of foods taken by each child at home were achieved by inquiring trained parents. When collecting the children’s dietary record form filled in by parents, investigators checked it carefully and would asked parents to supplement it in time if missing data was found. The weight of food intake at home was assessed based on the size of the food the parents filled in on the dietary record form. Finally, the dietary intake of calories and nutrients were calculated and analyzed by using the China Food Composition Tables [32, 33] for each day, and were presented the average intake of the 6-day period, including vitamin A and carotenoids. And the proportion of vitamin A from carotenoid sources were calculated. Intake of vitamin A was presented as retinol equivalents (RE) and retinol activity equivalents (RAE): 1 µg RE = 1 µg retinol = 6 µg β–carotene = 12 µg other provitamin A carotenoids, 1 µg RAE = 1 µg retinol = 12 µg β–carotene = 24 µg other provitamin A carotenoids [3]. Children with lower (< 0.07µmol/g) and higher (> 1.05µmol/g) liver vitamin A reserves were excluded when determining adequate intake levels.

### Quantification of vitamin A stores with the DRD assay

The isotope-labeled D_8_ vitamin A (Octadeuterated retinyl acetate (all-trans-retinyl- 10,14,19,19,19,20,20,20-[^2^H_8_] acetate) used in this study was synthesized by Cambridge Isotope Laboratories (Andover, MA), and dissolved in absolute ethanol and then corn oil. The ethanol was subsequently dried with nitrogen. On day 1, the isotope-labeled D_8_ vitamin A (2.5 mg/subject) was injected to a piece of cake designated for the breakfast of each child. The cake (100 g) and soy milk (200ml) were ingested under the monitoring of investigators to make sure that it is really ingested by the participants. On the day 3 or day 18, fasting blood samples were collected and serum was isolated and stored at -80℃. The subjects of 3-d group and 18-d group were different, for it was difficult to draw blood repeatedly from one child. Deuterated and nondeuterated retinol isotopes were analyzed firstly by separating retinol from the serum by using HPLC, then collecting the retinol fraction and drying by nitrogen, and derivatizing retinol into trimethylsilyl derivatives, which were then measured by using a gas chromatograph-electron capture negative chemical ionization mass spectrometer equipped with a 15 m DB-1 column and an on-column injector [31].

### Calculation of total and liver vitamin a stores by 3-d or 18-d DRD

Values of vitamin A reserves were expressed as total-body stores of vitamin A (mmol retinol) and as liver vitamin A concentration (µmol retinol/g liver). The vitamin A concentration in liver in the present study was estimated by assuming that liver weight is 3.0% of body weight and 90% of total-body vitamin A is storied in the liver [4, 10–14]. The vitamin A storage on day 18 was calculated with the following modified Bausch-Rietz Eqs. [16–27, 34]: Total body vitamin A store = F × dose × [s × α × (1/(D:H)-1)] (Eq. 1). F is a factor that represents efficiency of storage of an orally administered dose (0.5). Dose is the amount of labeled retinyl acetate administered (mmol). S (0.65) is the ratio of the specific activity of retinol in plasma versus liver. Factor a is a correction for the irreversible loss of vitamin A and is based on the half-life of vitamin A in liver, and the half-life was estimated to be 32 days in children [4] (a = e-kt, where k = 0.693/32). D:H is the ratio of deuterated (isotope-labeled) to non-deuterated (non-labeled) retinol. The “-1” is a term to correct the contribution of the dose of labeled vitamin A to the total vitamin A pool.

On 3-d DRD assay, the pseudo-equilibration of serum deuterated retinol over the non-deuterated retinol has not been reached yet, so the vitamin A store on day 3 was calculated differently. Based on Tang’s studies [29], the following equation can be used to evaluate the labeled vitamin A enrichment on day 21 with results from day 3: Predicted % enrichment on day 21 day = -0.061 + 0.243 × observed % enrichment on 3 days (Eq. 2), and then the modified Bausch-Rietz equation (Eq. 1) was used to calculate the total body stores of vitamin A.

### Statistical analyses

Student’s unpaired t test (for equal variances) or corrected t test (for unequal variances) were used to detect statistical significance of means between two groups. The correlations between vitamin A indices (serum retinol level, liver and total stores of vitamin A) and dietary vitamin A intake were examined by the partial correlation, age, gender, height, and weight were included as control variables. All statistical analyses were performed by using the IBM SPSS Statistics 26.0 software package. For all tests, a *P* value < 0.05 was considered statistically significant.

## Results

### Selected subjects represent typical well-nourished child

There were 38 boys and 22 girls among the selected 60 children. They were categorized into the 3-d group or 18-d group based on the day of blood sampling after the oral dose of the isotope-labeled vitamin A. Levels of body weight, height, BMI, serum albumin, serum CRP, and blood hemoglobin were all within the normal ranges (Table 1), and there were no significant difference in above indicators between 3-d group and 18-d group.

**Table 1:** Characteristics of the subjects^1^

	4 ~ 6 y			7 ~ 9 y			Total
	**3-d group** **(n = 12)**	**18-d group** **(n = 10)**	**P value**	**3-d group** **(n = 16)**	**18-d group** **(n = 22)**	***P*** **value**	**(n = 60)**
Height (cm)	110.4 ± 8.5	112.8 ± 5.5	0.47	127.8 ± 7.1	132.2 ± 5.1	0.07	123.4 ± 11.3
Weight (kg)	20.1 ± 4.1	21.4 ± 3.3	0.39	27.2 ± 5.1	30.7 ± 6.1	0.11	26.1 ± 6.6
BMI (kg/m^2^)	16.3 ± 1.0	16.8 ± 1.5	0.34	16.6 ± 1.9	17.4 ± 2.1	0.21	16.9 ± 1.8
Albumin (g/L)	48.4 ± 5.8	49.7 ± 2.0	0.52	50.2 ± 2.1	51.2 ± 3.4	0.34	50.1 ± 3.6
CRP (mg/L)	2.5 ± 0.9	2.2 ± 1.1	0.47	1.2 ± 0.3	1.1 ± 0.1	0.30	1.6 ± 0.9
Hemoglobin (g/dL)	118.2 ± 7.6	123.3 ± 12.2	0.24	118.4 ± 8.4	116.6 ± 5.0	0.41	118.5 ± 8.1

^1^ Data are presented as mean ± *SD*. There was no significant difference between the 3-day and 18-day groups in each age group

(Student’s unpaired t test, *P* > 0.05)

### Status of vitamin A of the selected subjects

The vitamin A status of the participants was shown in Table 2. There was no significant difference in vitamin A store in body and liver between 3-d group and 18-d group in each age group. The results show that the level of liver vitamin A on day 3 before equilibrium is reached can approximately reflect that of vitamin A on day 18 when equilibrium is reached.

**Table 2:** Vitamin A status of the subjects^1^

	4 ~ 6 y			7 ~ 9 y			Total
	**3-d group** **(n = 11)**	**18-d group** **(n = 10)**	**P value**	**3-d group** **(n = 16)**	**18-d group** **(n = 21)**	***P*** **value**	**(n = 58)**
Serum β-carotene (µmol/L)	0.56 ± 0.16	0.41 ± 0.16	0.63	0.39 ± 0.23	0.39 ± 0.22	0.94	0.43 ± 0.21
Serum retinol (µmol/L)	1.32 ± 0.13	1.31 ± 0.19	0.98	1.22 ± 0.31	1.26 ± 0.25	0.71	1.27 ± 0.24
Serum D:H^2^	0.006 ± 0.003	0.014 ± 0.012	0.07*	0.009 ± 0.007	0.009 ± 0.006	0.69	0.009 ± 0.007
Estimated total body vitamin A stores (mmol retinol)^3^	0.35 ± 0.23	0.22 ± 0.19	0.18	0.29 ± 0.22	0.25 ± 0.13	0.58*	0.28 ± 0.19
Estimated liver vitamin A (µmol/g liver)^4^	0.51 ± 0.29	0.31 ± 0.27	0.13	0.33 ± 0.28	0.26 ± 0.15	0.37*	0.33 ± 0.25

^1^ Data are presented as mean ± *SD*. Two samples were unresponsive in the detection of enrichment of labeled vitamin A, so n = 58. There was no significant difference between the 3-day and 18-day groups in each age group (Student’s unpaired t test for equal variances or corrected t test for unequal variances, *P* > 0.05. * the corrected P value)

^2^ D:H: the ratio of deuterated (isotope-labeled) to non-deuterated (non-labeled) retinol. In a deuterated-retinol-dilution (DRD) procedure, the serum samples collected from children on 3 day or 18 day after dosing of 2.5 mg [^2^H_8_] retinyl acetate (D_8_) were used to determine the enrichment of deuterated and non-deuterated retinol by using a gas chromatograph-electron capture-negative chemical ionization-mass spectrometry (Tang et al., 1998). For 3-day DRD before the equilibrium is reached, Tang’s method (2002) was used to predict the % enrichment of deuterated retinol on day 21 after the equilibrium is reached

^3^ The total body vitamin A reserves were calculated by the modified Bausch-Rietz equation

^4^ Liver vitamin A reserves were estimated by assuming that the weight of the liver is 3% of body weight and that 90% of total vitamin A is in the liver

### Partial correlations of dietary intakes with vitamin A status

**Fig. 1:**
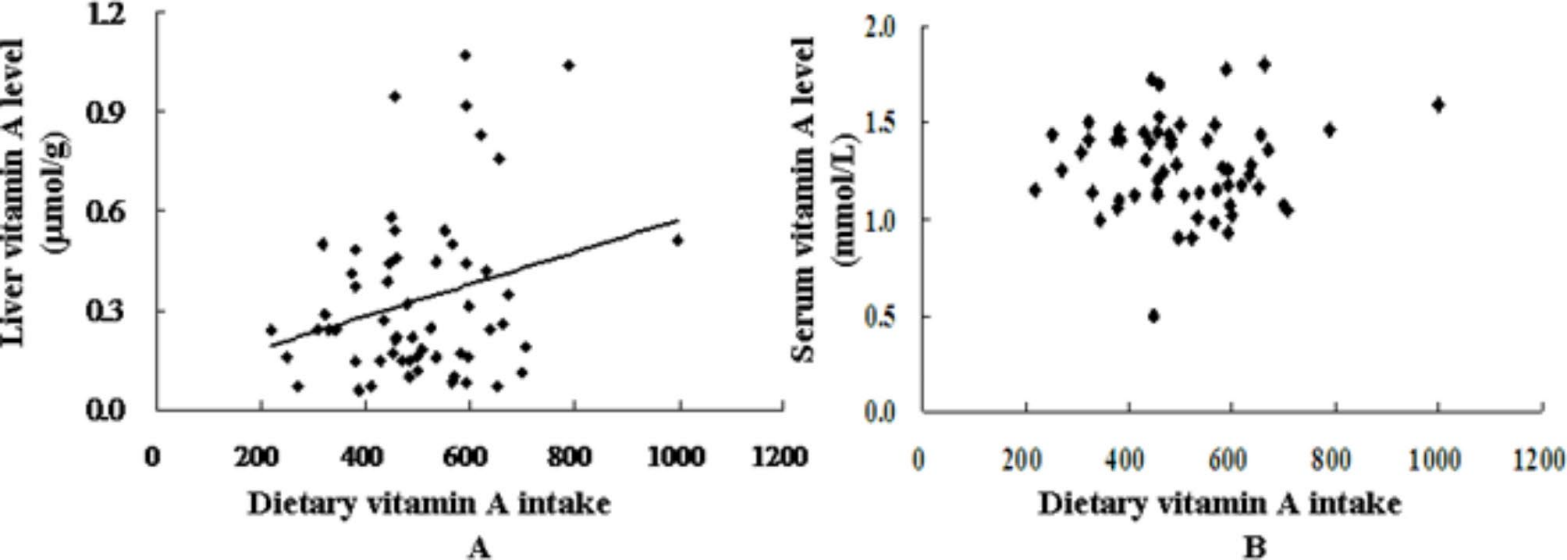
Scatter plots of the dietary vitamin A intake versus liver vitamin A stores (A) or serum levels (B). Liver vitamin A stores were estimated by using a deuterated-retinol-dilution procedure and calculated assuming that liver weight is 3.0% of body weight and 90% of total body vitamin A is in the liver. Serum vitamin A levels were determined by HPLC. Dietary vitamin A intakes were estimated from two consecutive 3-day weighed records and dietary recalls. Pearson correlation analysis was used to detect the correlations between dietary vitamin A intake and liver vitamin A stores (*r* = 0.270, *P* = 0.040) or serum vitamin A levels (*r* = 0.047, *P* = 0.721)

As shown in Table 3, after accounting for age, gender, height, and weight, there was a significant correlation between estimated dietary vitamin A intake and total body vitamin A store (for µg RE, *r* = 0.308, *P* = 0.025; for µg RAE, *r* = 0.280, *P* = 0.043) and liver vitamin A store (for µg RE, *r* = 0.349, *P* = 0.010; for µg RAE, *r* = 0.313, *P* = 0.022). It was noteworthy that vitamin A intake had no correlation with serum level of vitamin A (Fig. 1). Intake of β-carotene was also correlated with both total body vitamin A store (*r* = 0.322, *P* = 0.019) and liver stores of vitamin A (*r* = 0.382, *P* = 0.005), but not correlated with the serum level of vitamin A. The Bland-Altman plots were used to assess the level of agreement between the variables that were significantly correlated (*P* < 0.05) in Table 3, an additional file showed this in more detail [see Additional file 4].

**Table 3:** Partial correlations of dietary intakes with vitamin A status in the subjects^1^

Measured from blood samples	Estimated from dietary records	*r*	*P*
Total body vitamin A stores^2^	Vitamin A (µg RE)^3^	0.308	**0.025**
Total body vitamin A stores	Vitamin A (µg RAE)^4^	0.280	**0.043**
Total body vitamin A stores	β-Carotene (µg)	0.322	**0.019**
Total body vitamin A stores	Carbohydrate (g)	-0.039	0.781
Total body vitamin A stores	Protein (g)	0.027	0.847
Total body vitamin A stores	Total fat (g)	0.050	0.723
Liver vitamin A concentrations^5^	Vitamin A (µg RE)^3^	0.349	**0.010**
Liver vitamin A concentrations	Vitamin A (µg RAE)^4^	0.313	**0.022**
Liver vitamin A concentrations	β-Carotene (µg)	0.382	**0.005**
Liver vitamin A concentrations	Carbohydrate (g)	0.009	0.948
Liver vitamin A concentrations	Protein (g)	0.050	0.722
Liver vitamin A concentrations	Total fat (g)	0.058	0.681
Serum retinol concentrations	Vitamin A (µg RE)^3^	-0.068	0.631
Serum retinol concentrations	Vitamin A (µg RAE)^4^	-0.075	0.594
Serum retinol concentrations	β-Carotene (µg)	-0.013	0.928
Serum retinol concentrations	Carbohydrate (g)	-0.159	0.257
Serum retinol concentrations	Protein (g)	-0.283	**0.040**
Serum retinol concentrations	Total fat (g)	-0.217	0.119

^1^Partial correlation analysis coefficients (*r*) were calculated form 58 children (all variables were normal distribution when one-sample kolmogorov-smirnov test was used). Control variables were age, gender, height, and weight

^2^Total body vitamin A stores were assessed by using the deuterated-retinol-dilution procedure

^3^RE, retinol equivalents. Total amounts of dietary vitamin A intake were presented as RE, including the amount of retinol and carotenoids intake. RE (µg) = retinol (µg) + 1/6 β–carotene (µg) + 1/12 other carotenoids (µg)

^4^RAE, retinol activity equivalents. Total amounts of dietary vitamin A intake were presented as RAE, including the amount of retinol and carotenoids intake. RAE (µg) = retinol (µg) + 1/12 β–carotene (µg) + 1/24 other carotenoids (µg)

^5^Liver vitamin A concentrations were estimated by assuming that liver weight is 3.0% of body weight and 90% of total body vitamin A is in the liver

*P* values that less than 0.05 were bolded

### Recommended vitamin A intake in well-nourished chinese children

Two children with lower (< 0.07µmol/g) and one with higher (> 1.05µmol/g) liver vitamin A reserves were excluded. The calculated amounts of vitamin A intake among the subjects with a normal level of vitamin store in liver were 476.9 ± 196.7 µg RE/d or 377.7 ± 166.2 µg RAE/d (4–6 y) and 529.1 ± 87.2 µg RE/d or 464.0 ± 81.1 µg RAE/d (7–9 y) for vitamin A, and 1053.8 ± 518.2 µg/d (4–6 y) and 782.7 ± 224.6 µg/d (7–8 y) for β-Carotene (Table 4). Thus, we defined the estimated adequate dietary vitamin A intakes among well-nourished Chinese children were around 477 µg RE/d (95%CI 385 ~ 570) or 378 µg RAE/d (95%CI 304 ~ 441) for 4 ~ 6 y children and 529 µg RE/d (95%CI 500 ~ 560) or 464 µg RAE/d (95%CI 437 ~ 491) for 7 ~ 9 y children.

**Table 4:** Dietary intakes of vitamin A of the children with adequate liver vitamin A reserve^1^

	Age		
	**4 ~ 6 y (n = 20)**	**7 ~ 9 y (n = 35)**	**Total (n = 55)**
Vitamin A (µg RE /d)	476.9 ± 196.7	529.1 ± 87.2	507.4 ± 137.3
Vitamin A (µg RAE /d)	377.7 ± 166.2	464.0 ± 81.1	442.3 ± 123.4
β-carotene (µg/d)	1053.8 ± 518.2	782.7 ± 224.6	879.5 ± 376.9
vitamin A from plant foods (RE %)	36.8	24.7	28.9
vitamin A from plant foods (RAE%)	23.3	14.0	17.0

^1^ Data are presented as mean ± *SD*. (all variables were normal distribution when one-sample kolmogorov-smirnov test was used to test).The liver vitamin A levels of ≥ 0.07µmol/g and < 1.05 µmol/g liver are considered as adequate vitamin A reserve. Two children with lower (< 0.07umol/g) and one with higher (> 1.05umol/g) liver vitamin A reserve were excluded, two samples were unresponsive in the detection of enrichment of labeled vitamin A, so n = 55

## Discussion

Requirements of vitamin A have been studied for several decades, but the dietary requirement of vitamin A for Chinese children has not been established yet. In the present study, we estimated an adequate dietary vitamin A intake associated with sufficient liver vitamin A reserves among well-nourished Chinese children. Our results firstly provided an important data and bases to establish vitamin A requirements of this age group.

In our study, the vitamin A status of the children was measured by using serum retinol level first, and then further confirmed by RID technique. Consecutive 3-d weighed food records conducted in different seasons, which provided a reasonable estimate of dietary vitamin A intakes for the children. Our data also showed that dietary vitamin A or β-Carotene intakes were significantly correlated with total-body vitamin A or liver vitamin A levels, but not correlated with serum retinol concentrations, which were consistent with other previous studies [16, 26]. Therefore, it is feasible to estimate the dietary requirement based on the dietary intakes in children with adequate liver reserves (0.07–1.05 µmol /g liver) in the present study.

According to these data, we estimated the values of adequate dietary vitamin A intake were around 477 µg RE/d (95%CI 385 ~ 570) or 378 µg RAE/d (95%CI 304 ~ 441) for 4 ~ 6 y children and 529 µg RE/d (95%CI 500 ~ 560) or 464 µg RAE/d (95%CI 437 ~ 491) for 7 ~ 9 y children. The results are close to the current RNIs (Recommend) of Chinese children (360 µg RAE/d for the children aged 4–6 y, and 500 µg RAE/d for the children aged 7–11 y) [3], but lower than the RDAs of U.S. children (400 µg RAE/d for the children aged 4–8 y, and 600 µg RAE/d for the children aged 9–13 y) [6, 7]. We also compared the results with that of the other Asian countries, Japan (450 µg RE/d for the children aged 3–8 y, and 500 µg RE/d for the children aged 8–10 y) [35], and Korean (400 µg RE/d for the children aged 6–8 y, and 500 µg RE/d (girls), 550 µg RE/d (boys) for the children aged 9–11 y) [36, 37], and found that the results were relatively close.

Our finding of a significant correlation between dietary β-carotene with liver vitamin A or total-body vitamin A, has important implications for the effectiveness of plant carotenes as a vitamin A source. In view of carotenes have different important functions and have no toxicity as vitamin A, so in the long run, maybe we should establish the dietary reference intake of retinol and carotenes respectively. Furthermore, our study showed that there was a significant reverse correlation between dietary intakes of protein and serum retinal concentrations. However, since we did not observe a correlation between protein intake and liver vitamin A or total-body vitamin A, whereas serum retinol concentrations are controlled by homeostasis, we thought this might just be a concomitant relationship, but this view needs further evidence to support it.

Conventional DRD procedure needs 11 ~ 26 days of equilibration between serum deuterated vitamin A and the body’s vitamin A storage pool after dosing of deuterated vitamin A. A shorter term (such as 3 days) DRD become preferable in the field research because it help to decrease the isotope dilution by dietary vitamin A, reduce the loss of labeled vitamin A via catabolism and increase the compliance of subjects. Thus, several studies [28–30] have tried to develop 3-d DRD assays to estimate the equilibrated total body vitamin A stores, but their methods and results remain to be validated. In this study, we compared the results from day 3 to those from day 18 by using several different methods [21, 28–30]. The vitamin A reserve in both whole body and liver were similar between day 3 and day 18 when Tang’s method was used [29]. Since the 3-day group and the 18-day group were randomized sampling, there should be no statistical difference between the two groups, so this result was in line with expectations. Since our subjects and Tang’s subjects were of the same age, and they were all Chinese children, and all the experimental procedures were completed in the same laboratory. For these reasons, in the end, we took Tang’s method. However, we also need to realize that Tang’s method was a regression equation that can predict the 21-d enrichment data from the 3-d isotope enrichment data, rather than a direct prediction equation, which may not have good generalizability.

DRD has been used in other studies to obtain information on dietary vitamin A requirement. One study carried out in Filipino elders, Ribaya-Mercado et al. [16] investigated the dietary vitamin A intakes of men and women aged > 60y with adequate (≥ 0.07µmol/g) or low (< 0.07µmol/g) liver vitamin A concentrations determined by DRD. The dietary vitamin A intake was only 135 ± 95 µg RAE /d in well-nourished elders, which is much lower than that in well-nourished children in our study. In another study [20], paired DRD was used to estimate the changes of vitamin A pool size in Bangladeshi men before and after supplementation. Different from our study and Ribaya-Mercado’s study, this study controlled dietary vitamin A intake by providing low vitamin A diet and additional supplementing vitamin A. This approach is more reliable for estimating dietary requirement, but high cost and requiring better compliance of the subject limit its application in certain population, such as children. In addition to the DRD method, some researchers have attempted to use mathematical modeling methods to estimate VA total body store (TBS) and liver VA concentrations. Recently, Ford et al. [38] developed a mathematical relationship, defined by a series of equations, to predict changes in TBS as a function of age based on VA intake and disposal rate, the predictions were similar with published values. As more information becomes available, the mathematical relationship may provide a valuable tool for future work designed to assess dietary VA recommendations and the efficacy and safety of VA intervention strategies.

One important limitation of our study is that due to the cross-sectional nature of this study, it cannot be assumed that these children were in a steady state, or in positive/negative balance at the time of the study, so future cohort studies are needed to address this limitation. Besides, the mean dietary intake obtained by two consecutive 3-day weighting method in different seasons may not fully reflect the habitual intake of these children, in the future study, we will adopt the NCI method [39, 40] or try to carry out a non-consecutive dietary survey over 20 days to obtain a more accurate intake of vitamin A. Moreover, when processing dietary data, we focused on the average daily intake of energy and nutrients per child, especially vitamin A and carotenoids, and ignored the intake of various foods, this prevent us from having further discussions on certain issues. Such as what is the top contributors of plant foods to vitamin A intake among these children. We could consider reanalyzing our dietary data in the future. Nonetheless, this is the first study that assesses dietary intakes of vitamin A with objective measures of vitamin A reserves among well-nourished Chinese children, this study provides useful information on the evaluation of liver vitamin A reserve to establish the dietary requirement of vitamin A specifically for Chinese children.

## Conclusion

In the present study, we assessed the total-body and liver vitamin A reserves of children aged 4–9 y by use of the RID technique and determined their dietary vitamin A intakes from two consecutive 3-d weighed food records. On the basis of the data of the present study, we suggest the estimated values of adequate dietary vitamin A intake are around 477 µg RE/d (95%CI 385 ~ 570) or 378 µg RAE/d (95%CI 304 ~ 441) for 4 ~ 6 y children and 529 µg RE/d (95%CI 500 ~ 560) or 464 µg RAE/d (95%CI 437 ~ 491) for 7 ~ 9 y children. Although it needs to be verified in larger population of different regions in China, our results provide important data firstly based on evaluation of liver vitamin A reserve to establish dietary requirement of vitamin A specifically for Chinese children.

## Electronic supplementary material

Below is the link to the electronic supplementary material.

Supplementary Material 1

Supplementary Material 2

Supplementary Material 3

Supplementary Material 4

## Data Availability

The datasets used and analysed during the current study are available from the corresponding author on reasonable request.

## Declarations

### Clinical trial

NCT01559766, Registered 21 March 2012.

## Abbreviations

VAD: Vitamin A deficiency.
AI: Adequate intake.
RID: Retinol isotope dilution.
DRD: Deuterated retinol dilution.
CRP: C-reactive protein.
RE: Retinol equivalents.
