# Supplementary material for: Dietary vitamin A intakes of chinese children with adequate liver stores as assessed by the retinol isotope dilution technique
Source: BMC Pediatr. 2022 Oct 17;22:599. doi: 10.1186/s12887-022-03660-0 (PMC9575266; doi:10.1186/s12887-022-03660-0)
Supplement: Supplementary file 1 — Supplementary Material 1 [file 12887_2022_3660_MOESM1_ESM.docx]

**Supplementary Table 1** Characteristics of subjects that compared group 1 (n=123) and group 2 (n=60)^1^

|  | **Group1 (n=123)** | **Group2 (n=60)** | ***P* value** |
| --- | --- | --- | --- |
| Age (years) | 6.6±1.7 | 6.6±1.7 | 0.997 |
| Gender (male) | 69(56.1%) | 38(63.3%) | 0.491 |
| Height (cm) | 122.4±10.9 | 123.6±111.3 | 0.524 |
| Weight (kg) | 25.3±6.0 | 26.1±6.6 | 0.411 |
| BMI (kg/m^2^) | 16.6±1.7 | 16.8±1.8 | 0.467 |
| Albumin (g/L) | 49.5±5.0 | 50.1±3.6 | 0.417 |
| CRP (mg/L) | 1.6±0.9 | 1.6±0.9 | 0.964 |
| Hemoglobin (g/dL) | 120.0±8.7 | 127.9±51.7 | 0.242 |
| Serum retinol (μmol/L) | 1.27±0.1 | 1.28±0.2 | 0.781 |

^1^ Data are presented as mean ± *SD*. There was no significant difference between the group 1 (n=123) and group 2 (n=60).
