# Supplementary figures and images for: Dietary vitamin A intakes of chinese children with adequate liver stores as assessed by the retinol isotope dilution technique
Source: BMC Pediatr. 2022 Oct 17;22:599. doi: 10.1186/s12887-022-03660-0 (PMC9575266; doi:10.1186/s12887-022-03660-0)

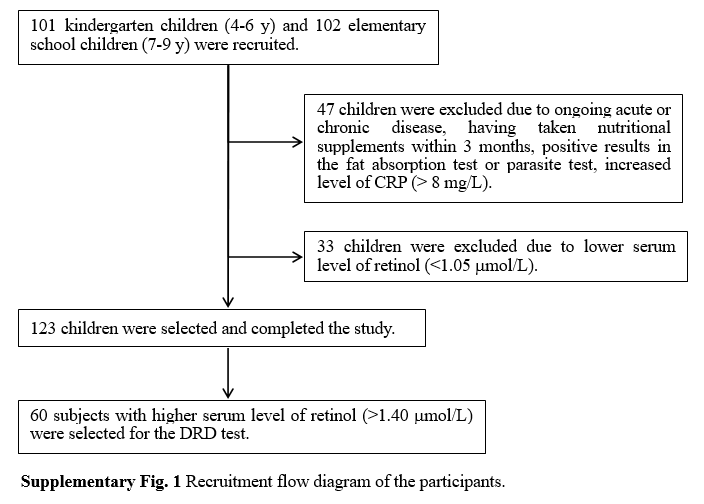

Supplement: Supplementary file 2 — Supplementary Material 2 [file 12887_2022_3660_MOESM2_ESM.doc]

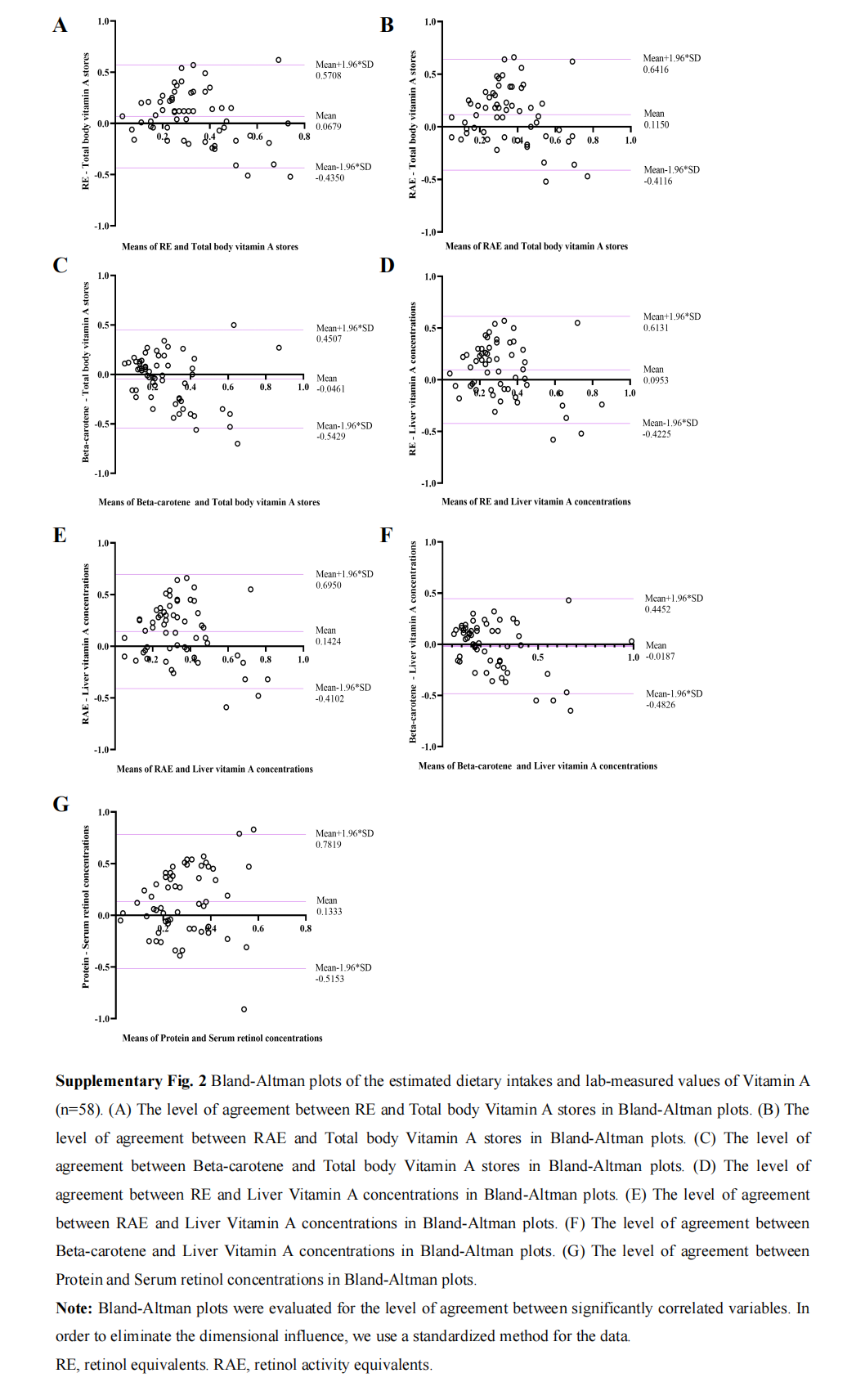

Supplement: Supplementary file 4 — Supplementary Material 4 [file 12887_2022_3660_MOESM4_ESM.doc]
