# Supplementary material for: Dietary vitamin A intakes of chinese children with adequate liver stores as assessed by the retinol isotope dilution technique
Source: BMC Pediatr. 2022 Oct 17;22:599. doi: 10.1186/s12887-022-03660-0 (PMC9575266; doi:10.1186/s12887-022-03660-0)
Supplement: Supplementary file 3 — Supplementary Material 3 [file 12887_2022_3660_MOESM3_ESM.docx]

**Supplementary Table 2** Comparison of the results of two dietary surveys^1^.

|  | **Winter** | **Summer** | ***P* value** |
| --- | --- | --- | --- |
| **Elementary school** |  |  |  |
| Vitamin A (μg RE /d) | 189.0±58.4 | 541.8±118.7 | <0.001 |
| Energy (kcal) | 1539.0±342.9 | 1392.8±239.6 | 0.023 |
| Protein (g) | 57.9±12.1 | 51.5±10.5 | 0.010 |
| Fat (g) | 55.7±11.8 | 68.3±13.3 | <0.001 |
| Carbohydrate (g) | 209.7±60.2 | 146.7±23.8 | <0.001 |
| **Kindergarten** |  |  |  |
| Vitamin A (μg RE /d) | 338.9±127.7 | 446.1±170.7 | 0.003 |
| Energy (kcal) | 1150.9±243.7 | 1312.2±270.9 | 0.008 |
| Protein (g) | 47.3±9.1 | 55.9±13.9 | 0.002 |
| Fat (g) | 37.8±9.3 | 44.7±12.9 | 0.010 |
| Carbohydrate (g) | 158.9±33.9 | 178.5±36.2 | 0.018 |

^1^ Data are presented as mean ± *SD*. There were significant differences between the winter and summer (*P*<0.05).
